# Inhibitory Effect of Indigo Naturalis on Tumor Necrosis Factor-α-Induced Vascular Cell Adhesion Molecule-1 Expression in Human Umbilical Vein Endothelial Cells

**DOI:** 10.3390/molecules15096423

**Published:** 2010-09-14

**Authors:** Hsin-Ning Chang, Jong-Hwei Su Pang, Sien-Hung Yang, Chi-Feng Hung, Chi-Hsin Chiang, Tung-Yi Lin, Yin-Ku Lin

**Affiliations:** 1 Department of Traditional Chinese Medicine, Chang Gung Memorial Hospital, 222 Mai Chin Road, Keelung 204, Taiwan; E-Mail: 8705015@adm.cgmh.org.tw (H.N.C.); dryang@ adm.cgmh.org.tw (S.H.Y.); tungyi30@adm.cgmh.org.tw (T.Y.L.); 2 College of Medicine, Chang Gung University, 259 Wen-Hwa 1st Road, Kweishan, Taoyuan 333, Taiwan; E-Mail: jonghwei@mail.cgu.edu.tw (J.H.S.P.); 3 School of Medicine, Fu Jen Catholic University; Taipei County 242, Taiwan; E-Mail:skin@mails.fju.edu.tw; 4 Department of Obstetrics and Gynecology, Chang Gung Memorial Hospital, Keelung, 204, Taiwan; E-Mail: g61110126@cgmh.org.tw

**Keywords:** psoriasis, indigo naturalis, human umbilical vein endothelial cell, vascular cell adhesion-1, c-Jun

## Abstract

The use of indigo naturalis to treat psoriasis has proved effective in our previous clinical studies. The present study was designed to examine the anti-inflammatory effect of indigo naturalis in primary cultured human umbilical vein endothelial cells (HUVECs). Pretreatment of cells with indigo naturalis extract attenuated TNF-α-induced increase in Jurkat T cell adhesion to HUVECs as well as decreased the protein and messenger (m)RNA expression levels of vascular cell adhesion molecule-1 (VCAM-1) on HUVECs. Indigo naturalis extract also inhibited the protein expression of activator protein-1 (AP-1)/c-Jun, a critical transcription factor for the activation of VCAM-1 gene expression. Since the reduction of lymphocyte adhesion to vascular cells by indigo naturalis extract could subsequently reduce the inflammatory reactions caused by lymphocyte infiltration in the epidermal layer and help to improve psoriasis, this study provides a potential mechanism for the anti-inflammatory therapeutic effect of indigo naturalis extract in psoriasis.

## 1. Introduction

Psoriasis is an immune-mediated inflammatory skin disease and the transport of leukocytes from the dermis into the epidermis is a key event in psoriasis [1]. It has been proposed that migration of leukocytes from the circulatory system to skin, via interactions with endothelial cells of blood vessels mediated by adhesion molecules, is pivotal event in the pathogenesis of psoriasis [2]. Previous studies have been demonstrated that the expression of intercellular adhesion molecule-1 (ICAM-1) and vascular cell adhesion molecule-1 (VCAM-1) are increased in dermal vessels of psoriatic lesions [3]. VCAM-1 on endothelium is critical for initial trafficking of memory T cells in psoriatic plaques, while ICAM-1 enhances the infiltration of T cells [4]. The increased expressions of ICAM-1 and VCAM-1 on dermal vessels in psoriatic lesions have been demonstrated, at least in part, due to the overproduction of tumor necrosis factor-α (TNF-α) in psoriatic skin lesions [5]. Thus, TNF-α plays an important role in activating the expression of adhesion molecules on vascular endothelial cells and then causes the subsequent increased trafficking of leukocytes in psoriasis. Additionally, production of TNF-α is activated through several transcription factors, including Activator protein 1 (AP-1), and nuclear factor-κB (NF-κB) [6,7]. Blocking the generation of an inflammatory infiltrate by interfering with key molecules of the adhesion process is an attractive strategy to treat psoriasis; numerous approved drugs, such as Efalizumab, use this approach [8].

Recently, the use of traditional Chinese herbal medicines (CHMs) for the treatment of psoriasis has attracted much attention from patients and physicians [9]. We have reported that an ointment based on indigo naturalis as effective at reducing psoriasis symptoms [10,11]. Indigo naturalis (QingDai) is a dark blue powder extracted from the leaves of indigo-bearing plants, such as *Baphicacanthus cusia* Bremek, *Polygonum tinctorium*, *Isatis indigotica* and *Strobilanthes formosanus* Moore (Acanthaceae). Until the mid-twentieth century, when antibiotics and steroids came into the world, indigo naturalis was one of the most important traditional CHMs used to heal various human ailments resulting from infections and inflammatory diseases in China and Taiwan. For decades indigo naturalis has been successfully used to treat leukemia and psoriasis [12,13]. However, the precise mechanism of action of indigo naturalis on psoriasis is unclear. Our previous histological results showed significant pathological improvement accompanied by decreasing expressions of Ki-67, CD3-positive T cells, in combination with the restoration of filaggrin after topical indigo naturalis treatment, and *in vitro* studies also demonstrated that indigo naturalis modulates differentiation and proliferation in cultured human keratinocytes [14,15]. To the best of our knowledge, the effects of indigo naturalis on the pro-inflammatory cytokine-stimulated adhesion process have not yet been clarified. To clarify the mechanism by which indigo naturalis is effective against psoriasis, the present study was designed to focus on the enhanced expression of cell adhesion molecules on dermal vessels in psoriatic skin. We studied the effects of indigo naturalis on TNF-α-stimulated human umbilical vein endothelial cells (HUVEC) characterizing their T cells adherence and their expression of ICAM-1 and VCAM-1. The effects of indigo naturalis on TNF-α-activated transcription factors were also determined.

## 2. Results and Discussion

### 2.1. Indigo Naturalis Extract Inhibited TNF-α-induced Adhesion of Jurkat T cells to HUVECs

**Figure 1:**
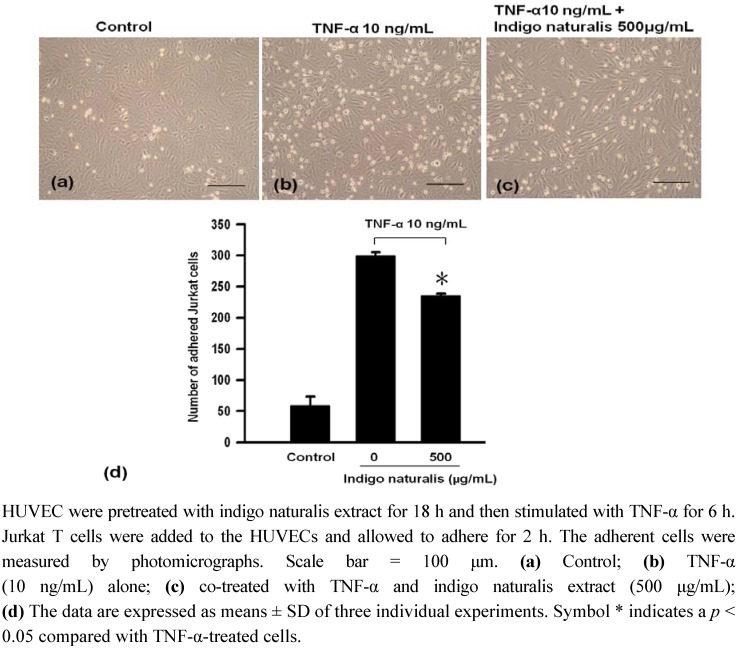
Effect of indigo naturalis extract on Jurkat T cells adhesion to HUVEC.

The biological consequence of TNF-α-induced adhesion molecule expression on HUVEC cell surface was determined using a quantitative cellular adhesion assay with Jurkat T cells. The adhesion of Jurkat T cells to HUVEC was markedly up-regulated following treatment by TNF-α for 6 h (Figure 1a b ). A decrease in the adherence of Jurkat T cells to HUVEC was observed following pretreatment of HUVEC with 500 μg/mL indigo naturalis extract (Figure 1c. This data indicates that treatment of HUVECwith indigo naturalis extract blocked about 26.4% TNF-α-induced Jurkat-T cell adhesion to HUVEC (Figure 1d). This inhibitory effect was not due to cytotoxicity because culturing with 500 μg/mL indigo naturalis extract for 24 h did not cause lactate dehydrogenase (LDH) release (data not shown).

### 2.2. Indigo Naturalis Extract Inhibits TNF-α- induced VCAM-1 Gene Expression in HUVECs

**Figure 2:**
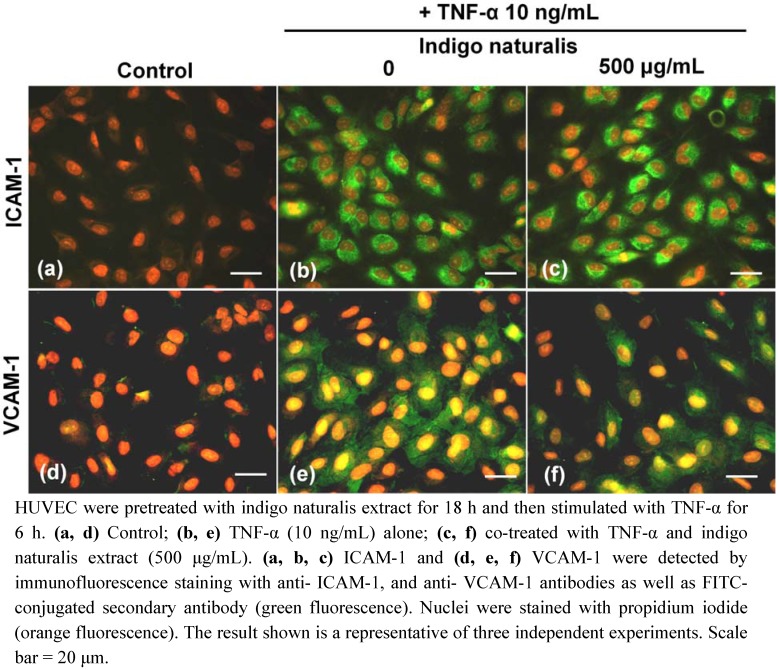
Effect of indigo naturalis extract on TNF-α induced expression of adhesion molecules.

To characterize the molecular mechanisms responsible for the down-regulation of TNF-α-induced Jurkat T cell adhesion to HUVEC by indigo naturalis extract, we used immunofluorescence staining, real-time RT-PCR and Western blot analysis to determine the level of ICAM-1 and/or VCAM-1 expression in HUVEC. Confluent cultures of HUVEC pretreated with or without indigo naturalis extract for 18 h and then stimulated with TNF-α for 6 h were analyzed for ICAM-1 and for VCAM-1. Results from the immunofluorescent analysis showed that ICAM-1 and VCAM-1 expression (green fluorescence) were markedly enhanced over the cytoplasm after stimulation of TNF-α (Figure 2b, e), however, only VCAM-1 expression was markedly inhibited after indigo naturals extract treatment (Figure 2f). In the control group, HUVEC showed negative or scattered faint staining for ICAM-1 and VCAM-1 (Figures 2a d 

**Figure 3:**
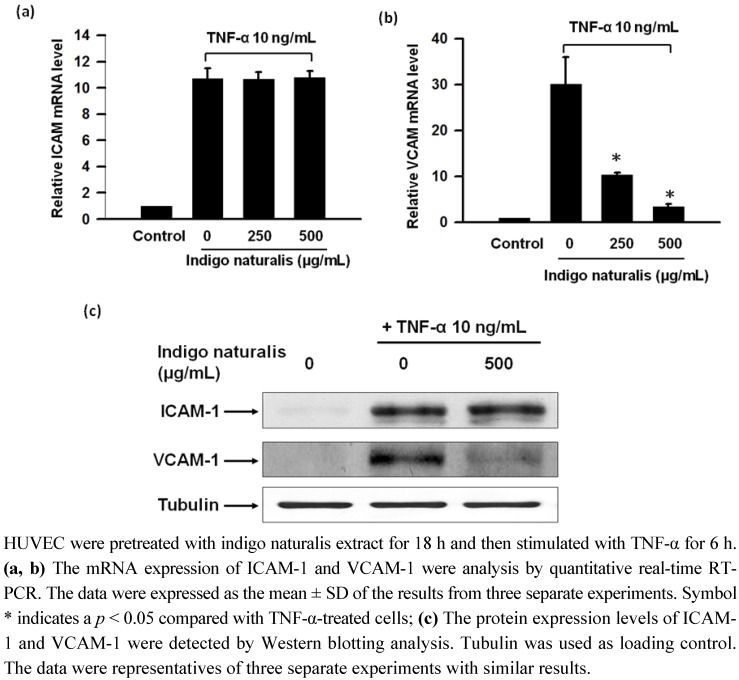
Effect of indigo naturalis extract on TNF-α induced mRNA and protein expression of adhesion molecules.

In results from the real-time RT-PCR and Western blot analysis, both ICAM-1 and VCAM-1 were almost undetectable without TNF-α treatment and were significantly increased after 6 h of treatment of TNF-α (Figures 3a-c ). Indigo naturalis extract treatment clearly abrogated the stimulation of VCAM-1 expression elicited by TNF-α and ICAM-1 expression was slightly enhanced by indigo naturalis extract, which were consistent with the results of immunofluorescence staining shown in Figure 2. The effect of indigo naturalis extract inhibited inducible VCAM-1 mRNA and protein expression revealed a dose-dependent connection (Figure 3b).

### 2.4. Indigo Naturalis Extract Inhibits TNF-α-induced c-Jun Expression in HUVECs

**Figure 4:**
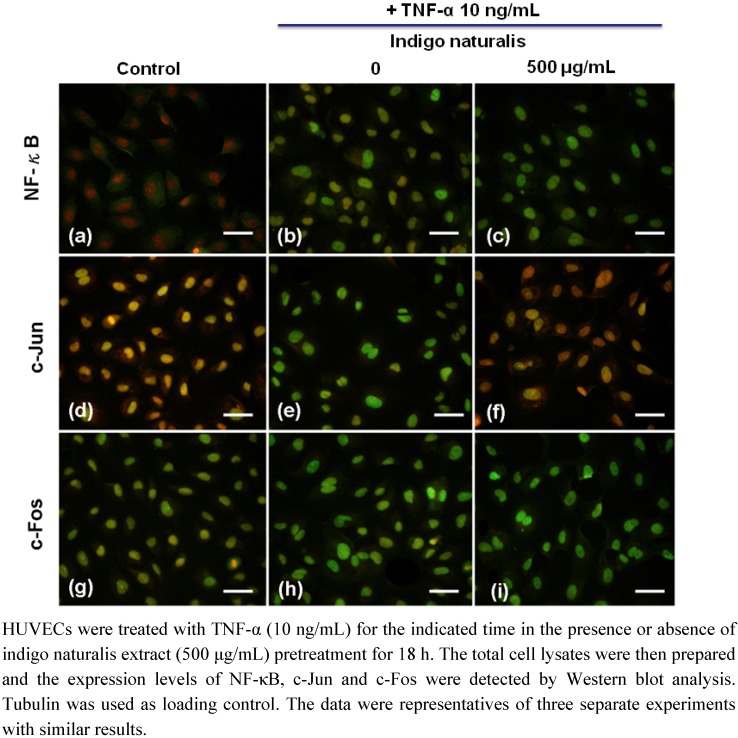
Effect of indigo naturalis extract on protein expression of NF-κB, c-Jun and c-Fos on HUVECs.

The binding motif of NF-κB and AP-1(c-Jun/c-Fos) has been identified in ICAM-1 and VCAM-1 promoters and shown to be involved in the induction of ICAM-1/VCAM-1 gene expression. To evaluate whether the inhibitory effect of indigo naturalis extract on inducible VCAM-1 expression was mediated by a NF-κB- or AP-1(c-Jun/c-Fos)-dependent pathway, the same methods were used as described above. TNF-α treatment for 60 min significantly increased NF-κB, c-Jun and c-Fos gene expression in HUVEC as measured by immunofluorescence staining (Figures 4a-i . Among these transcription factors, only c-Jun expression markedly decreased in indigo naturalis extract-pretreated-HUVEC (Figure 4f).

**Figure 5:**
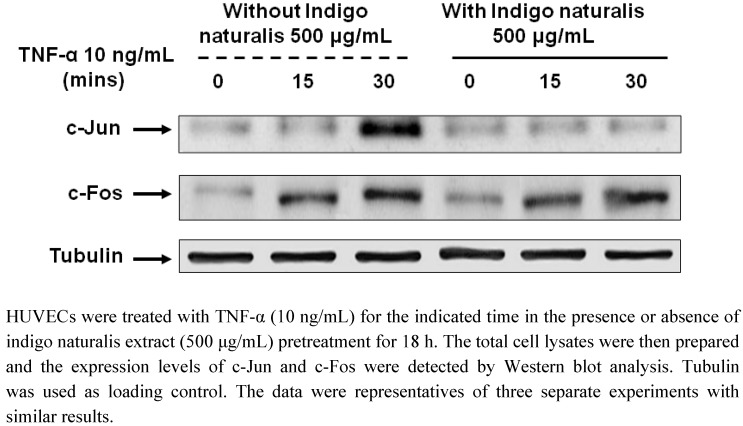
Effect of indigo naturalis extract on protein expression of c-Jun and c-Fos on HUVECs.

In addition, when the time-course protein expression of c-Jun induced by TNF-α in HUVECs was analyzed by Western blotting, the result showed significantly decreased inducible c-Jun protein expression in indigo naturalis-pretreated HUVEC at 30 min (Figure 5). This result again proves that AP-1/c-Jun plays a role in the suppression of indigo naturalis extract on TNF-α stimulated HUVECs as indicated by immunofluorescent staining shown in Figure 4. We have previously reported evidence-based research results showing that the topical application of indigo naturalis can significantly improve psoriatic symptoms [16]. This randomized, observer-blind, vehicle-controlled, intrapatient comparison study has attracted much attention from western mainstream newspapers and medical magazines.

Moreover, the previous studies also demonstrated that indigo naturalis modulates differentiation and proliferation in epidermal keratinocytes as well as inhibits human neutrophil proinflammatory responses [15,17]. In this present study, we first showed that treatment with indigo naturalis extract significantly attenuated T cell adhesion to TNF-α-stimulated HUVEC by inhibiting VCAM-1 expression at both protein and mRNA levels. Additionally, indigo naturalis inhibits TNF-α-induced VCAM-1 expression which may be closely related to the inhibition of AP-1/c-Jun activation. These findings suggest that indigo naturalis’ anti-psoriatic effect may be partially mediated by suppressing vascular inflammation and the expression of VCAM-1 by vascular endothelial cells.

Recently, there have been a number of studies investigating the effects of anti-psoriatic agents upon the expression of cell adhesion molecules. *In* *vivo* studies demonstrated the decreased expression of ICAM-1 and VCAM-1 on the dermal vessels with improvement of skin lesions following treatment with Methotrexate (MTX), PUVA and UVB radiation [18,19]. *In vitro*, MTX was also demonstrated to inhibit the TNF-α induced ICAM-1 and VCAM-1 expression on HUVEC [20]. Retinoic acid inhibits the TNF-α-induced VCAM-1 expression, but not ICAM-1 expression on human dermal microvascular endothelial cells [21]. These studies suggest that the beneficial effect of agents in treatment of psoriasis may be associated with the reduction of cell adhesion molecules on dermal vessels. In our previous *in vivo* studies the T cell infiltrate was reduced after indigo naturalis ointment therapy, as well as the expression of the VCAM-1 (unpublished data). This *in vitro* study shows that indigo naturalis extract can down-regulate TNF-α-induced VCAM-1 gene expression, independent of ICAM-1. Interestingly, VCAM-1 plays a major role in TNF-α induced adhesion of Jurkat cells to HUVEC contributing to about 60% of such adhesion [22]. Taken together, it suggests that indigo naturalis therapy and current anti-psoriatic therapy may share some common working mechanisms, which involved the inhibition of cell adhesion molecule expression on vascular endothelium.

Transcription factors are another target of current therapies for psoriasis, such as topical vitamin D derivatives and retinoid, which indirectly target AP-1 and NF-κB signaling [23]. It has been demonstrated that TNF-α induces VCAM-1 gene expression in endothelial cells through activating the transcription factor NF-κB and AP-1 [24]. In our experiment, indigo naturalis extract blocked the TNF-α-activation of c-Jun expression, independent from NF-κB in HUVEC. AP-1 is a transcriptional regulator that functions as a regulator of cytokine expression and an important modulator in inflammatory diseases, such as psoriasis [25]. Previous studies showed while c-Fos protein expression appears unchanged in normal and psoriatic epidermis, c-Jun expression is enhanced in psoriatic epidermis and dermis [26,27]. It has been speculated that up-regulation of c-Jun in basal keratinocytes might contribute to the pathogenesis. Additionally, the increased c-Jun expression in psoriasis might be activated by the increased TNF-α production in this disease [28]. Although our data showed indigo naturalis suppressed TNF-α-induced c-Jun expression on HUVEC, the effect of indigo naturalis on cytokine signaling associated with proliferation in human epidermal keratinocytes is unexplored. Further investigation is warranted as to whether the modulating effect of indigo naturalis in proliferation and differentiation of psoriasis is through inhibiting c-Jun gene expression.

We recognize our study was limited in that the effect of the individual active components of indigo naturalis, such as indirubin and tryptanthrin, was not fully explored. However, it is difficult in Chinese herb studies to use the modern drug discovery method based on the lock-and-key theory, which attempts to use one single compound to hit one target to combat the related disease [29]. In fact, the multicomponent therapeutic strategy, which has received much attention in recent years, has many advantages over single component strategy, such as it may exerts synergistic effects and exerts effects at low concentration, potentially yielding increased safety over single-component drugs [30]. In our preliminary experiments using adhesion assays showed that all three major components of indigo naturalis, indigo, indirubin and tryptanthrin exerted similar effects on block T cell adhere to TNF induced HUVEC (data not shown). However, not any one of them can individually exert the total pharmacological function of indigo naturalis. This result suggests that these components of indigo naturalis may act synergistically. Taken together, the synergistic effects of these different components may be required for the entire anti-psoriatic efficacy of indigo naturalis.

## 3. Experimental

### 3.1. Materials

Indigo naturalis powder was purchased from Guang Sheng Trading (Taipei, Taiwan), and was prepared from the plant *Strobilanthes formosanus* Moore (Acanthaceae). The plant was grown in the mountains near Sansia, Taiwan, and was identified by Dr. Rong-Chi Yang, the chief of the Chinese Herbal Pharmacy at Chang Gung Memorial Hospital, Taoyuan, Taiwan. A voucher specimen (SF-1) is deposited in the herbarium of Chang Gung University, Taoyuan, Taiwan. The indigo naturalis powder was dissolved in dimethyl sulfoxide (DMSO) in a proportion of 1:10 (w/v), then sterilized by filtration (pore size 0.2 μm), and stored at −20 °C for subsequent bioassay testing. The quantity analysis of standard samples by Dr. Yann-Lii Leu found 0.701 mg/mL indirubin, 0.387 mg/mL indigo, and 0.044 mg/mL tryptanthrin in the extract.

### 3.2. Cell Culture

Primary HUVECs were isolated by collagenase digestion of the interior of the umbilical vein from human umbilical cord obtained from Chang Gung Memorial Hospital. HUVECs were cultured in M199 medium supplemented with 20% fetal calf serum (FCS), 200 μg/mL Endothelial Cell Growth Factor Supplement (ECGF, Sigma, St. Louis, MO, USA) and 100 U/mL penicillin-100 μg/mL streptomycin (Gibco BRL, Life Technologies, Grand Island, NY, USA) at 37 °C in a humidified incubator containing 5% CO_2_. Cells were trypsinized when they reached 80-90% confluence and were passaged into gelatin-coated flasks. Cells were used for the experiment from the first three to five passages. Human Jurkat T cells colon E6-1 (American Type Culture Collection, ATCC, Bethesda, MD, USA) were cultured in RPMI 1640 medium supplemented with 10% fetal bovine serum and 100 U/mL penicillin-100 μg/mL streptomycin. Cells were maintained at 37 °C in a humidified incubator containing 5% CO2. Indigo naturalis extract stock solutions for cell treatment were prepared in DMSO at concentrations such that the final concentration of the solvent in cell suspension never exceeded 0.3% (v/v). Respective controls were treated with equal volume of DMSO.

### 3.3. Cell Cytotoxicity Test

Cell cytotoxicity was measured using LDH release assay. Cell death leads to release of cytoplasmic proteins into the culture supernatant. LDH release was determined by a commercially available method (Promega, Madison, WI, USA). Fluorescence was recorded at a wavelength of 492 nm using a Dynex Technologies MRX^TM^ (Washington D.C., USA). As positive control, the total LDH activity was determined by lysing cells with 0.1% Triton X-100 for 30 min at 37 °C. Cytotoxicity was expressed as the percent LDH activity obtained in cell-free medium compared to the total LDH activity.

### 3.4. Adhesion of Jurkat T cell to HUVEC

Monolayers of HUVEC were plated at 5 × 10^4^ cells per well in gelatin-coated 6-well plates. After 24 h of seeding, HUVEC were pretreated with of 500 μg/mL indigo naturalis extract for 18 h, and then activated with 10 ng/mL TNF-α for 6 h. HUVEC wells were washed with PBS and then co-cultured with Jurkat T cells (2 × 10^6^ cells/mL) for 30 min in a culture incubator with humidified air containing 5% CO_2_ at 37 °C. Indigo naturalis extract was not present in the incubation medium during co-culture period. Following 30 min of co-culture, non-adherent Jurkat-T-overlayed HUVECs were washed. Finally, photographs of five fields were taken from each well under 100 × magnification and adherence cell numbers were counted manually.

### 3.5. Immunofluorescence Staining

Confluent cultures of HUVEC on gelatin-coated coverslips in six-well plates were incubated with or without 500 μg/mL of indigo naturalis extract for 18 h at 37 °C. Then 10 ng/mL TNF-α was added without changing the culture media. After indicated time incubation, the cell were washed with PBS, fixed in 10% formaldehyde for 15 min at room temperature and permeabilized with 100% methyl alcohol for 10 min at −20 °C. Staining was performed in subsequent steps. Briefly, samples were blocked in 1% BSA and 1% goat serum for 30 min at room temperature followed by dilution of the primary antibodies: anti-ICAM-1 (1:100; mouse; Santa Cruz, CA, USA), anti-VCAM-1 (1:100; rabbit; Santa Cruz, CA, USA), anti-c-Jun (1:100; rabbit; Cell Signaling Technology, Beverly, MA, USA), anti-c-Fos (1:50; mouse, Santa Cruz, CA, USA), and anti-NF-κB (1:100; mouse; Santa Cruz, CA, USA), and then incubated for 1 h. Next, dilution of the secondary antibodies, fluorescein isothiocyanate (FITC)-conjugated goat anti-mouse/rabbit antibodies (1:150 in PBS; Leinco Technologies, Ballwin, MO, USA), was followed by incubation for 30 min at room temperature, another washing and propidium iodide (PI) staining. Finally, the coverslips were mounted with fluorescent mounting medium (DakoCytomation). A Nikon DXM1200 microscope and Nikon ACT-1 image analysis software were used for data processing.

### 3.6. Quantitative Real-time RT-PCR Analysis

The cells were treated with or without 500 μg/mL indigo naturalis for 18 h, and stimulated with10 ng/mL TNF-α for 6 h. Total RNA was extracted from endothelial cells using solution D (1 mL solution D/10^7^ cells). Subsequently, total RNA was extracted with phenol and chloroform: isoamyl alcohol (49:1) to remove proteins and genomic DNAs. Complementary (c) DNA was synthesized using 1 mg total RNA in a 20 mL volume RT reaction mix containing 0.5 mg of random primers,0.8 mM dNTP, 0.1 M DTT and 1 × first strand buffer.). Quantitative real-time RT-PCR was performed using an SYBR Green and MxPro- M × 3000P QPCR machine (Stratagene). Aliquots (20 ng) of cDNA were used for each quantitative PCR, and each reaction was run in triplicate. Relative gene expressions between experimental groups were determined using MxPro software (Stratagene) and 18S ribosomal RNA (rRNA) was used as an internal control. The following primers were used: ICAM-1: 5’-GCAAGAAGATAGCCAACCA-3’ (forward) and 5’-TGCCAGTTCCACCCGTTC- 3’ (reverse), VCAM-1: 5’-CATGACCTGTTCCAGCGAGG-3’ (forward) and 5’-CATTCACGAGGCCACCACTC-3’ (reverse), 18S: 5’-GTCTGCCCTATCAACT-3’ (forward), and 5’-TCGTCACTACCTCCC-3’ (reverse). All real-time PCRs were performed in triplicate, and changes in gene expressions were reported as multiples of increases relative to the untreated controls.

### 3.7. Western Blot Analysis

Cells were rinsed twice with ice-cold PBS and harvested in a lysis buffer (20 mM HEPES, 20 mM NaF, 1 mM Na_3_VO_4_, 1 mM Na_4_P_2_O_7_, 1 mM EDTA, 1 mM EGTA,1 mM DTT, 0.5 mM PMSF,1 μg/mL leupeptin and 1% Triton X-100) followed by sonication. Lysates were centrifuged at 13,200 × g for20 min, and the supernatants were retained. The protein concentration of the cell extracts was determined by the Bradford assay (Bio-Rad Laboratories, Richmond, CA). Samples containing 10-20 μg protein were boiled in sodium dodecyl sulfate (SDS) sample buffer and separated by 10%SDS-polyacrylamide gel electrophoresis (PAGE). Gels were blotted overnight onto polyvinylidenedifluoride (PVDF) membranes. Membranes were blocked with 1% BSA and 1% goat serum in PBS for 30 min at room temperature. After washing in PBS, membranes were incubated with the primary antibodies: anti-ICAM-1 (mouse), anti-VCAM-1 (rabbit), anti-c-Jun (rabbit) and anti-c-Fos (mouse) at an appropriate dilution (1:1000, 1:500, 1:400, 1:200 respectively) for 2 h at room temperature, and further incubated with goat anti-rabbit/mouse immunoglobulin G (IgG) conjugated with HRP-conjugated secondary antibodies (Cell Signaling, Danvers, MA, USA) for 1 h. Anti-tubulin antibody (1:1000) was used as the sample loading control. The protein bands were confirmed using the enhanced chemiluminescence reagent (Amersham Pharmacia Biotech, Little Chalfont, UK).

### 3.8. Statistical Analysis

Data are expressed as the mean ± SD. Statistical analysis of group differences was performed using Student’s t-test. A value of p < 0.05 was considered statistically significant.

## 4. Conclusions

The present data suggest that indigo naturalis could suppress TNF-α-induced VCAM-1 expression via inhibition of AP-1/c-Jun activation in primary cultured HUVEC, a phenomenon that may explain the decreased infiltration of T cells in indigo naturalis treatment contributing to its anti-inflammatory therapeutic effect in psoriasis. Our hope is that this understanding of its mechanism will lead scientific confirmation of anecdotal and historical accounts of the effectiveness of indigo naturalis against psoriasis and other inflammatory skin diseases.
